# THE ASSOCIATION BETWEEN RESIDENT ENGAGEMENT AND QUALITY OF CARE INTERACTION AMONG ACUTE CARE PATIENTS WITH DEMENTIA

**DOI:** 10.1093/geroni/igad104.1111

**Published:** 2023-12-21

**Authors:** Rachel McPherson

**Affiliations:** University of Maryland, Baltimore, Maryland, United States

## Abstract

Care interactions are essential to patients with dementia. The purpose of the study was to describe the quality of staff-patient care interactions among acute care (AC) patients with dementia and to test whether the quality of staff-patient care interactions varied by resident level of engagement in the interaction. Specifically, it was hypothesized that controlling for age, cognition, pain, behavior, and function, actively engaged patients would have more positive care interactions with staff compared with passively engaged patients. This secondary data analysis used baseline data from the Function Focused Care for Acute Care (FFC-AC- EIT), an ongoing intervention study. The sample included 286 patients. Descriptive statistics and a multiple linear regression were conducted. The majority of interactions that occurred were positive care (n = 225, 79%). Actively engaged patients had significantly more positive interactions compared to passively engaged patients (b = 1.49, p < .001). Patients with more pain (b = -0.26, p < .001) and behavioral symptoms (b = -0.12, p < .05) had significantly poorer care interactions. Passively engaged patients or patients with pain and behavioral symptoms may be at risk to receive poor quality care interactions. Findings from this study will inform future interventions and training for AC staff to promote positive care interactions among patients with dementia. Managing pain, managing behavior, and supporting resident engagement in care will likely help increase the frequency of positive care interactions in future work.

